# GOLPH3 induces epithelial–mesenchymal transition via Wnt/*β*‐catenin signaling pathway in epithelial ovarian cancer

**DOI:** 10.1002/cam4.1040

**Published:** 2017-03-23

**Authors:** Jing Sun, Xiaoming Yang, Ru Zhang, Suqing Liu, Xupei Gan, Xiaowei Xi, Zhenbo Zhang, Youji Feng, Yunyan Sun

**Affiliations:** ^1^ Department of Obstetrics and GynecologyShanghai General HospitalShanghai Jiao Tong University School of MedicineShanghaiChina

**Keywords:** Epithelial ovarian cancer, epithelial–mesenchymal transition, *GOLPH3*, metastasis, Wnt signaling pathway

## Abstract

*Golgi phosphoprotein 3* (*GOLPH3*), a newly recognized oncogene, is associated with tumor growth, metastasis, and poor prognosis in several types of cancer. However, its biological role and underlying mechanism in epithelial ovarian cancer (EOC) remain poorly understood. Here, we found that *GOLPH3* was overexpressed in EOC tissues and cell lines. This overexpression promoted the migration and invasion of EOC cells. Moreover, *GOLPH3* upregulated the expression of epithelial–mesenchymal transition (EMT) markers, such as *N‐cadherin* and *Snail*, and the Wnt/*β*‐catenin‐related genes *cyclin‐D1* and *c‐Myc*, which were restored via silencing of *GOLPH3* expression. Furthermore, the inhibitor and activator of the Wnt/*β*‐catenin pathway, XAV939 and LiCl, enhanced or decreased, respectively, the effect of *GOLPH3* on EMT, which further confirmed that *GOLPH3* promoted EMT progression via activation of Wnt/*β*‐catenin signaling. In addition, we found that *EDD*, the human hyperplastic discs gene, was consistent with *GOLPH3* expression and also promoted the EMT process and activated Wnt/*β*‐catenin signaling. These findings demonstrate that *EDD* might be a downstream factor of *GOLPH3*. Taken together, our findings demonstrate the existence of a GOLPH3–Wnt/*β*‐catenin–EMT axis in EOC and provide a new therapeutic target to treat EOC.

## Introduction

Ovarian cancer (OC) is a lethal gynecological malignant tumor and ranks fifth in cause of death for female cancer patients. In 2015, there were 14,180 deaths and 21,290 patients newly diagnosed with OC in the United States. However, the expected 5‐year survival rate of OC is only 45% 1. Of all reported OC subtypes, approximately 90% are epithelial ovarian cancer (EOC), which usually is present at advanced stage 2. For most advanced stage patients, cytoreductive surgery followed by platinum/taxol chemotherapy is regarded as the standard therapy 3. Although the majority of patients are sensitive to this treatment, over the past two decades, the overall cure rate hovers around 30% 4. The lack of effective early detection markers and tumor metastasis are major factors for poor outcomes and high death rates. Thus, improving targeting therapies and studying the mechanisms underlying tumor invasion and metastasis are necessary for reducing the OC mortality.

*Golgi phosphoprotein 3* (*GOLPH3*), also known as *GPP34/GMx33/MIDAS*, is a newly identified 34‐kDa phosphorylated matrix protein, which localizes to the transface of the Golgi complex and plays a critical role in the Golgi secretory pathway and DNA damage 5, 6, 7, 8, 9. *GOLPH3* resides on human chromosome 5p13, where it is amplified in multiple tumor types 10. Recent studies indicate that *GOLPH3* is involved in cancer progression and correlates with clinical stages and poor prognosis in several types of tumors 11, 12, 13, 14, 15. It has been reported that high *GOLPH3* expression promotes tumorigenicity and aggressive behavior of EOC 16, 17. However, its role in cell migration and invasion, as well as the molecular mechanism, remains unclear.

Metastasis is a process in which cancer cells spread from the primary tissue to surrounding tissues because cells lose cell–cell adhesion ability and gain migratory and invasive capability. Epithelial–mesenchymal transition (EMT) is defined as a dynamic process in which epithelial cells acquire the mesenchymal phenotype, which has motile and invasive characteristics 18. In recent years, accumulating evidence suggests that EMT is a crucial step in the cancer‐related metastatic cascade 19, 20. Various signaling pathways regulate EMT, including the HGF, EGF, TGF‐*β*, Notch, and Wnt/*β*‐catenin signaling pathways 19. As an important regulator of EMT, activation of the Wnt/*β*‐catenin pathway is common in many malignant tumors including EOC 21, 22, 23. Furthermore, it is proposed that the Wnt secretory pathway depends on endosome‐to‐Golgi transport 24, 25. However, the molecular mechanism of *GOLPH3* in cancer process, especially in migration and invasion, remains poorly understood. As a “first‐in‐class Golgi oncoprotein,” we speculate that *GOLPH3* may regulate Wnt/*β*‐catenin signaling pathway to promote EMT process in EOC.

Here, we provide evidences to confirm that overexpression of *GOLPH3* stimulates EMT via the Wnt/*β*‐catenin signaling pathway, which further promotes metastasis of EOC. In addition, we find that *EDD*, as a DNA damage‐related factor, and oncogene, like *GOLPH3*, might play an important role in GOLPH3–Wnt/*β*‐catenin–EMT axis of EOC. To our knowledge, these findings indicate the molecular mechanisms of *GOLPH3*‐mediated oncogenesis in EOC for the first time.

## Materials and Methods

### Patient information and tissue samples

The study was approved by medical ethical committee of Shanghai General Hospital affiliated to Medical School of Shanghai Jiao Tong University. All patients in our study provided written informed consent. In total, 58 EOC tissues and 15 benign tumor tissues were acquired from patients treated at our hospital between January 2014 and December 2016. Two gynecological pathologists confirmed all collected tissues based on the WHO classification.

### Immunohistochemistry

Paraffin‐embedded tissues were cut into 4 *μ*m sections. The immunohistochemistry (IHC) procedure to determine *GOLPH3* and *EDD* expression was performed as described previously 17. Briefly, the sections were incubated with mouse monoclonal anti‐*GOLPH3* antibody (1:200 dilution; Proteintech, Chicago, IL) and rabbit monoclonal anti‐*EDD* antibody (1:1000 dilution; Abcam, Cambridge, UK) overnight at 4°C. Negative control slides replaced the primary antibody with phosphate‐buffered saline (PBS). To detect the antigen, sections were incubated with biotinylated anti‐mouse or anti‐rabbit secondary antibody. Slides were evaluated at 200× magnification, and 10 different staining fields of each section were assessed independently by two trained observers who were blinded to patient information. A score criteria was assigned to evaluate the percentage of positively stained carcinoma cells, as previously reported 26.

### Cell culture

Human epithelial ovarian cancer cell lines, including HEY, SKOV3, HO8910, HO8910‐PM, and ES‐2 cell lines, were purchased from The Cell Bank of Type Culture Collection of the Chinese Academy of Sciences (Shanghai, China). The normal ovarian cell line (MOODY) was kindly provided by Dr. Wenxin Zheng (Department of Pathology, University of Texas Southwestern Medical Center, USA). All of the cells were grown in DMEM/F‐12 supplemented with 10% FBS and cultured in a sterile incubator maintained with 5% CO_2_ at 37°C.

### Western blot analysis

The western blot procedure was performed as described previously 27. Briefly, treated cells were lysed in RIPA lysis buffer containing protease inhibitor (1:1000). Approximately 30 *μ*g of the protein samples was separated by 7.5–12.5% SDS‐PAGE gels and then transferred to PVDF membranes (Millipore, Bedford, MA). After being blocked in 5% skim milk at room temperature for 1 h, the membranes were incubated with the corresponding specific primary antibodies (1:1000 dilution) overnight at 4°C. Then, the bands were robed with the appropriate secondary antibody (1:5000 dilution; Proteintech, Chicago, IL) at room temperature for 1 h. Enhanced chemiluminescence reagents ((Pierce, Rockford, IL) were used to detect antibody complexes. The primary antibodies used in our study included *GOLPH3*,* EDD* (Abcam, Cambridge, UK), *cyclin‐D1*,* c‐Myc* (Proteintech, Chicago, IL), *E‐cadherin*,* N‐cadherin*,* Snail*, and *β‐catenin* (Cell Signaling Technology, Danvers, MA). To determine the effect of Wnt/*β*‐catenin signaling, the pathway agonist LiCl (20 mmol/L; Sigma, St. Louis) and antagonist XAV939 (10 *μ*mol/L; Sigma) were used to treat cells for 24 h after transfection. *β‐actin* (Proteintech, Chicago, IL) was used as a loading control. Each experiment was performed in triplicate.

### Transient transfection

Cells were transiently transfected using Lipofectamine 2000 (Invitrogen, Grand Island, NY) following the manufacturer's protocol. Briefly, cells were seeded into six‐well plates at a density of 2 × 10^4^ cells/well. When cultured to 50–60% confluency, cells were serum starved for 24 h to minimize the influence of FBS. Then, cells were transfected with siRNA or plasmid using Lipofectamine 2000. After 6–8 h of incubation, the treated cells were cultured in DMEM/F‐12 with 10% FBS. The *GOLPH3* siRNA and negative control were constructed by GeneChen (Shanghai, China). The pcDNA3.1‐GOLPH3 and pcDNA3.1‐vector plasmids were designed and purchased from Genera Biotechnology (Shanghai, China). The sequences of the *GOLPH3* and *EDD* siRNA are listed in Table 1.

**Table 1:** GOLPH3 and EDD siRNA sequences

Name	Sequence
GOLPH3	
Sense	5′‐GGUGUAUUGACAACAGAGA‐3′
Antisense	5′‐UCUCUGUUGUCAAUACACC‐3′
EDD	
Sense	5′‐GCGUGAACGUGAAUCCGUU‐3′
Antisense	5′‐AACGGAUUCACGUUCACGC‐3′

### Quantitative real‐time PCR analysis

Total RNA was extracted from cells using Trizol Reagent (Invitrogen), following the manufacturer's protocol. Reverse transcription was performed in a 20‐*μ*L reaction system with 1 *μ*g of total RNA via the Prime‐Script RT reagent kit (Takara, Kyoto, Japan). The complimentary DNA (cDNA) was then synthesized with SYBR Premix Ex Taq (Takara, Dalian, China) for quantitative real‐time PCR. *β‐actin* served as the internal control gene. The amplification was performed for 40 cycles including 5 min at 95°C, 5 sec at 95°C, and 30 sec at 60°C. The data were analyzed using the 2^−ΔΔCT^ method to determine the relative gene expression levels. Each experiment was repeated three independent times. The PCR primers for *GOLPH3*,* EDD*, and *β‐actin* were synthesized by Sangon Biotech (Shanghai, China) and are listed in Table 2.

**Table 2:** PCR primer sequences

Name	Sequence
GOLPH3	
Forward primer	5′‐ACC TGT TTT GGG TTT CTG GT‐3′
Reverse primer	5′‐TGT GCG TAT GAG GAG GCT G‐3′
EDD	
Forward primer	5′‐CCA TAC AAA CGA CGA CGG T‐3′
Reverse primer	5′‐GCC AAC AGG AAC ATT CTT GAC‐3′
*β*‐actin	
Forward primer	5′‐AAG GTG ACA GCA GTC GGT T‐3′
Reverse primer	5′‐TGT GTG GAC TTG GGA GAG G‐3′

### Cell invasion and migration assays

Cell migration and invasion were assayed using Transwell plates with 8‐*μ*m pore filters (Corning, NY). The procedure was performed according to the manufacturer's protocol. Briefly, for the cell migration assay, transfected HEY, SKOV3, and HO8910 (2 × 10^5^) cells were suspended in 200 *μ*l of serum‐free DMEM and plated into the upper chambers. Then, 600 *μ*l of DMEM/F‐12 supplemented with 10% FBS was added to the lower chamber. After incubation for 12–14 h at 37°C, the tumor cells were fixed with 4% cold paraformaldehyde and stained with crystal violet (Beyotime, Shanghai, China). For the invasion assay, the procedures were conducted as described earlier, except the filter inserts were coated with BD Matrigel and the plates were incubated for 16–20 h at 37°C. Cells that had migrated were counted at 100× magnification under an inverted microscope.

### Scratch migration assay

For the scratch migration assay, treated cells were seeded onto six‐well plates to form cell monolayer (near 90% confluence). Subsequently, the cell layer was scratched with a 200‐*μ*L pipette tip, washed three times with PBS to remove floating cells, and photographed (time 0 h). Later, the wounded cultures were incubated in conditioned medium at 37°C. At 0, 24, and 48 h, images were captured using an inverted microscope to assess wound closure and then compared to determine differences in cell migration. Three fields (×100) were randomly selected from each scratch wound.

### Statistical analysis

Statistical analyses were performed using the Statistical Package for the Social Sciences (SPSS) 20.0 (IBM, Armonk, NY). The data are presented as the mean ± standard deviation (SD). The statistical difference between the test and control group was assessed using Student's *t*‐test. The association between *GOLPH3* and *EDD* expression was analyzed by using Spearman's correlation analysis. A *P* < 0.05 was considered statistically significant.

## Results

### High *GOLPH3* expression in epithelial ovarian cancer cells and tissues

**Figure 1:**
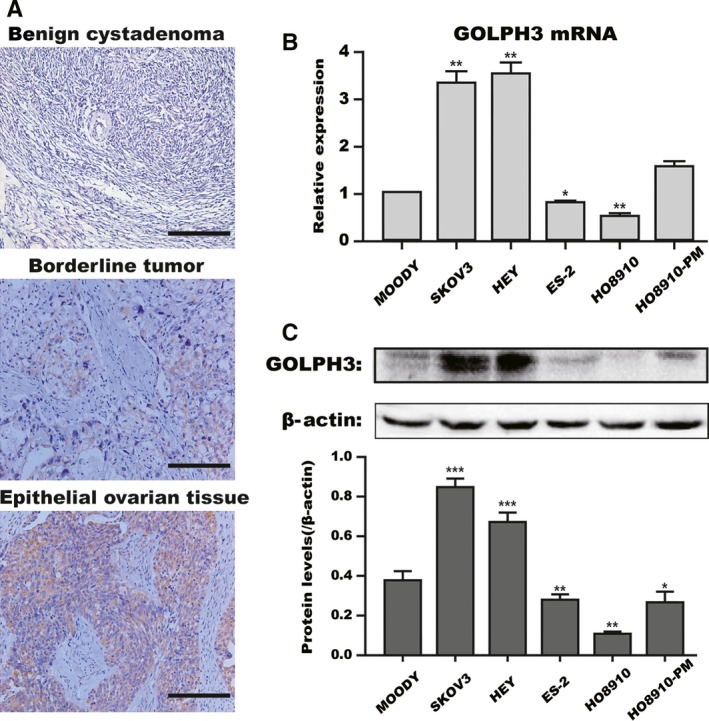
High GOLPH3 expression in epithelial ovarian cancer cells and tissues. (A) Immunohistochemical staining showed that GOLPH3 expression was higher in epithelial ovarian tissues than in benign cystadenoma and borderline tumors. Scale bar: 200 *μ*m. Original magnification 200×. GOLPH3 mRNA (B) and protein (C) expression in five ovarian cancer cell lines (HEY, SKOV3, ES‐2, HO8910, HO8910‐PM) and one normal ovarian cell line (Moody) were detected by quantitative real‐time PCR and western blot analyses. *β*‐Actin served as a loading control. The data were expressed as the mean ± SD of at least three independent experiments. *P <* *0.05, **P <* *0.01, **^*^ *P *<* *0.001 compared with control cells.

To investigate the oncogenic role of *GOLPH3* during EOC progression, we examined the expression level of *GOLPH3* in EOC tissues and cell lines. As shown in Table 3, ovarian tissue samples from 73 patients were used in this study. There were 2 (13.33%) of the 15 cases of benign tumors, 3 (60%) of the five cases of borderline tumors, and 45 (84.91%) of the 53 cases of epithelial ovarian cancer that showed high expression of *GOLPH3* protein. Clearly, *GOLPH3* expression was higher in epithelial ovarian cancer than in the borderline tumors and benign cystadenomas (Fig. 1A, *P* < 0.05).

**Table 3:** GOLPH3 expression in different ovarian tissues

Tissue type	Total	High level (%)	Low level	*P* value
Benign tumor	15	2 (13.33)	13	
Borderline tumor	5	3 (60.00)	2	<0.05^1^
Epithelial cancer	53	45 (84.91)	8	<0.05^2^

An IHC score of <3 was considered low expression and a score of 4–12 was considered high expression.

^1^Benign ovarian tumor versus borderline ovarian tumor.

^2^Benign ovarian tumor versus epithelial ovarian cancer.

In addition, to further explore the potential effect of *GOLPH3* in EOC, we measured the *GOLPH3* mRNA and protein expression in ovarian cell lines (Fig. 1B and C). As our data shows, *GOLPH3* was upregulated in two of five ovarian cancer cell lines (SKOV3 and HEY) at both the mRNA and protein level compared with the normal immortalized cell line, Moody (*P* < 0.05). These findings suggest that *GOLPH3* is overexpressed in human ovarian carcinoma and may contribute to some of the tumor behaviors.

### *GOLPH3* promotes the migration and invasion capacity of ovarian cancer cells

**Figure 2:**
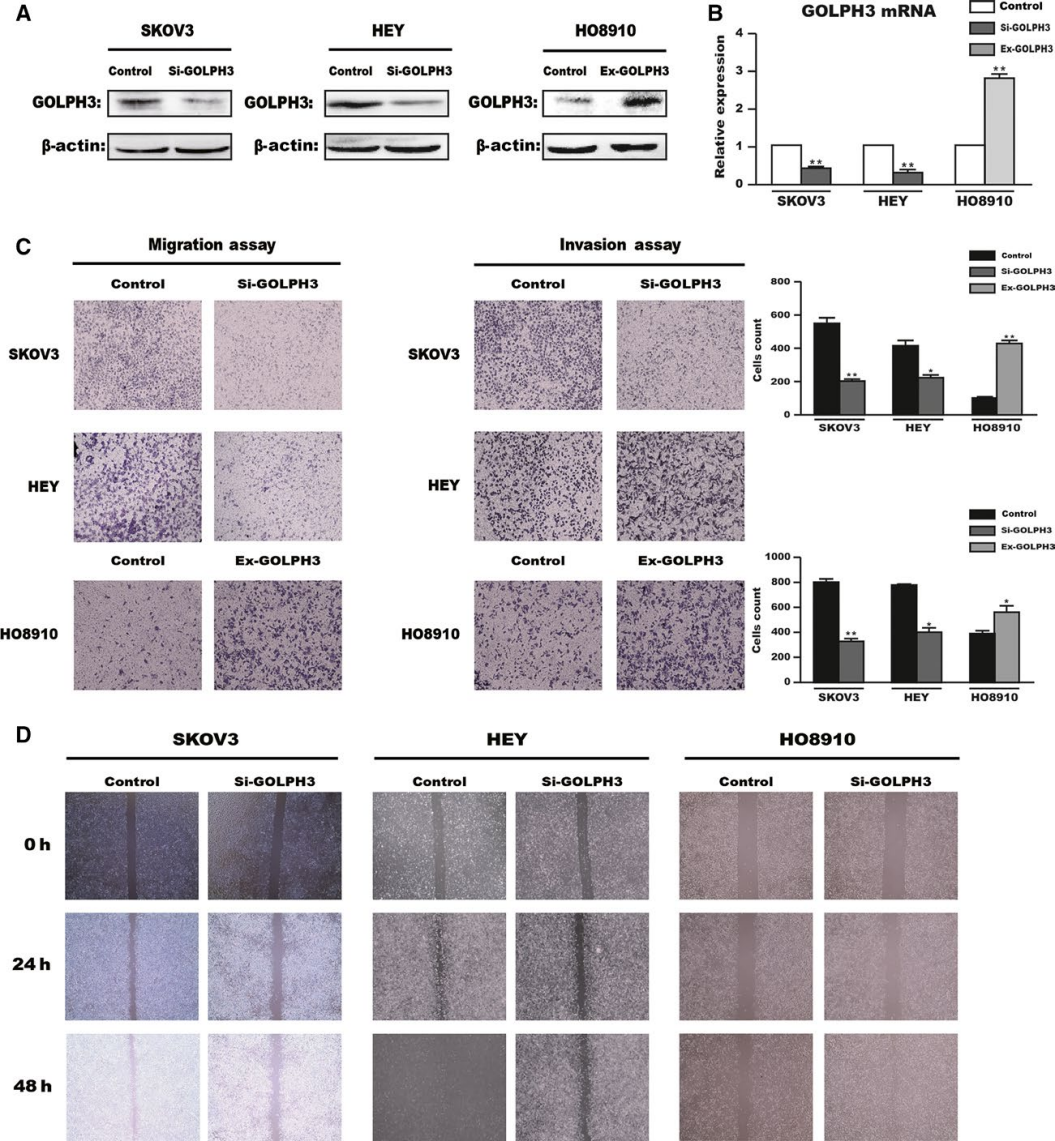
GOLPH3 promotes the migration and invasion capacity of ovarian cancer cells. GOLPH3 expression was downregulated by siRNA in HEY and SKOV3 cell lines and overexpressed by plasmid in the HO8910 cell line. The transfection efficiencies were measured by western blot (A) and quantitative real‐time PCR (B) analyses. *β*‐Actin served as a loading control. Transwell (C) and scratch migration (D) analyses were used to determine the function of GOLPH3 in the migratory and invasive capability of EOC cells. Original magnification 100×. *P <* *0.05, **P <* *0.01 compared with the control group.

Notably, we found that SKOV3 and HEY had a higher level of *GOLPH3* expression than the other cell lines, whereas HO8910 had the lowest expression. To examine the role of *GOLPH3* in EOC, we knocked down its expression in SKOV3 and HEY cells and overexpressed it in HO8910 cells. As our data shows, compared to the control group, the relative expression of *GOLPH3* mRNA and protein was significantly decreased in the siRNA‐transfected group and increased in the plasmid‐treated group (*P* < 0.01, Fig. 2A and B).

We then studied the impact of *GOLPH3* on cell migration and invasion in vitro. The results of the Transwell assay revealed that silencing *GOLPH3* significantly reduced the number of cells on the membrane filters for control, while overexpressing *GOLPH3* increased the number of cells present (*P* < 0.05, Fig. 2C). Moreover, scratch migration assays also showed that knockdown of *GOLPH3* in SKOV3 and HEY cells resulted in reduced wound healing ability compared with control cells, which was restored by increasing *GOLPH3* expression in HO8910 cells (Fig. 2D). Collectively, these results suggest that *GOLPH3* contributes to the migration and invasion capacity of EOC cells.

### *GOLPH3* is involved in EMT‐related protein expression in vitro

**Figure 3:**
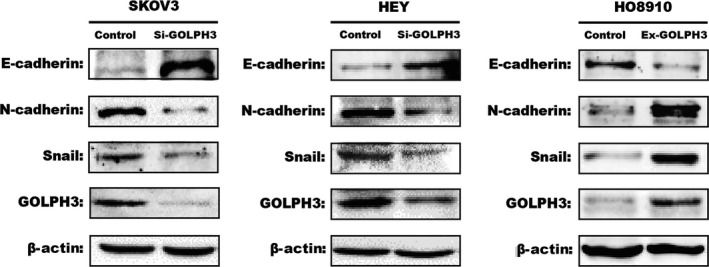
GOLPH3 induces EMT through the Wnt/*β*‐catenin signaling pathway. Silencing GOLPH3 expression in HEY and SKOV3 cell lines increased the expression of E‐cadherin and decreased N‐cadherin and Snail expression, which was restored via increasing GOLPH3 expression in HO8910 cell line. *β*‐Actin was served as the loading control.

Collectively, to further support the proposal that *GOLPH3* plays an important role in the migration and invasion of EOC cells, we examined the expression of EMT‐related proteins in treated cells through western blot analysis. As shown in Figure 3, compared to the control group, knockdown of *GOLPH3* in HEY and SKOV3 cells inhibited the expression of the mesenchymal marker, *N‐cadherin*, and stimulated the expression of the epithelial marker, *E‐cadherin*, and the EMT‐associated transcription factor, *Snail*, which was in contrast to *GOLPH3* overexpression in HO8910 cells. These results indicate that *GOLPH3* promotes migration and invasion via EMT in vitro.

### *GOLPH3* induces EMT through the Wnt/*β*‐catenin signaling pathway

**Figure 4:**
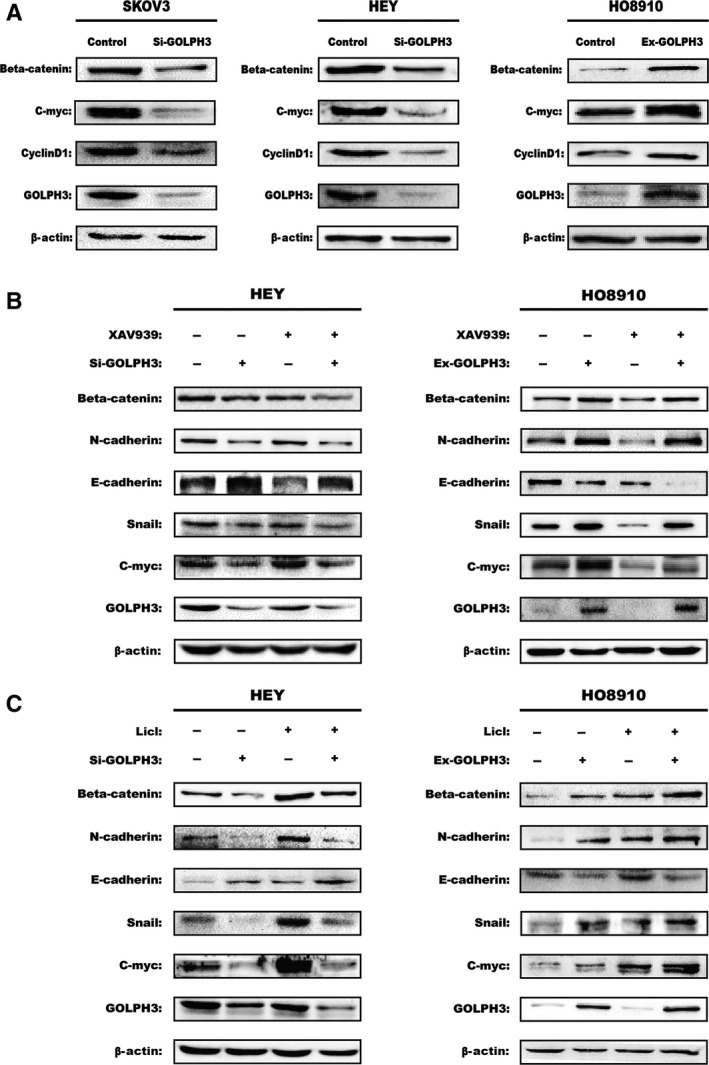
GOLPH3 induces EMT through the Wnt/*β*‐catenin signaling pathway. (A) Western blot analysis showed that silencing GOLPH3 expression in HEY and SKOV3 cell lines decreased *β*‐catenin, cyclin D1, and C‐myc expression, which was restored via increasing GOLPH3 expression in HO8910 cell line. Expression levels of GOLPH3, EMT‐related markers, and Wnt/*β*‐catenin target genes in the different cells following treatment with 10 *μ*mol/L XAV939 (B) or 20 mmol/L LiCl (C) for 24 h were measured using western blotting. The *β*‐actin was used as the loading control.

Due to the important role of the Wnt/*β*‐catenin signaling pathway in tumorigenesis and metastasis of tumors, we wondered whether GOLPH3 participated in the EMT process by activating Wnt/*β*‐catenin signaling. To address this question, we examined the expression of *β‐catenin*,* c‐Myc*, and *cyclin‐D1*, which are related to Wnt/*β*‐catenin signaling. In our results, we found that GOLPH3 overexpression in HO8910 cells significantly increased the expression *of β‐catenin*,* c‐Myc*, and *cyclin‐D1*. Conversely, knockdown of *GOLPH3* in HEY and SKOV3 cells suppressed the expression of these genes (Fig. 4A). The results above demonstrate that the Wnt/*β*‐catenin signaling pathway plays a functional role in *GOLPH3*‐induced EMT in EOC.

Moreover, the hypothesis was further confirmed by treating cells (HEY and HO8910) with the pathway‐specific antagonist XAV939 and agonist LiCl. In HEY and HO8910 cell lines, XAV939 significantly inhibited Wnt/*β*‐catenin signaling as manifested by the reduced expression of *β‐catenin* and *c‐Myc*, while LiCl activated this pathway (Fig. 4B and C). Moreover, we found that the effect on EMT‐related proteins was reversed by inhibiting the Wnt/*β*‐catenin pathway in *GOLPH3* overexpression group, while it was promoted by activating the pathway in *GOLPH3* interference group. Furthermore, as we expected, after adding XAV939 in *GOLPH3* interference group, the expression of EMT‐related proteins were further decreased, while these proteins were further increased by adding LiCl in *GOLPH3* overexpression group. Taken together, we concluded that *GOLPH3* induces EMT through the Wnt/*β*‐catenin signaling pathway in EOC.

### *GOLPH3* might regulate *EDD* during Wnt signaling‐induced EMT in EOC cells

**Figure 5:**
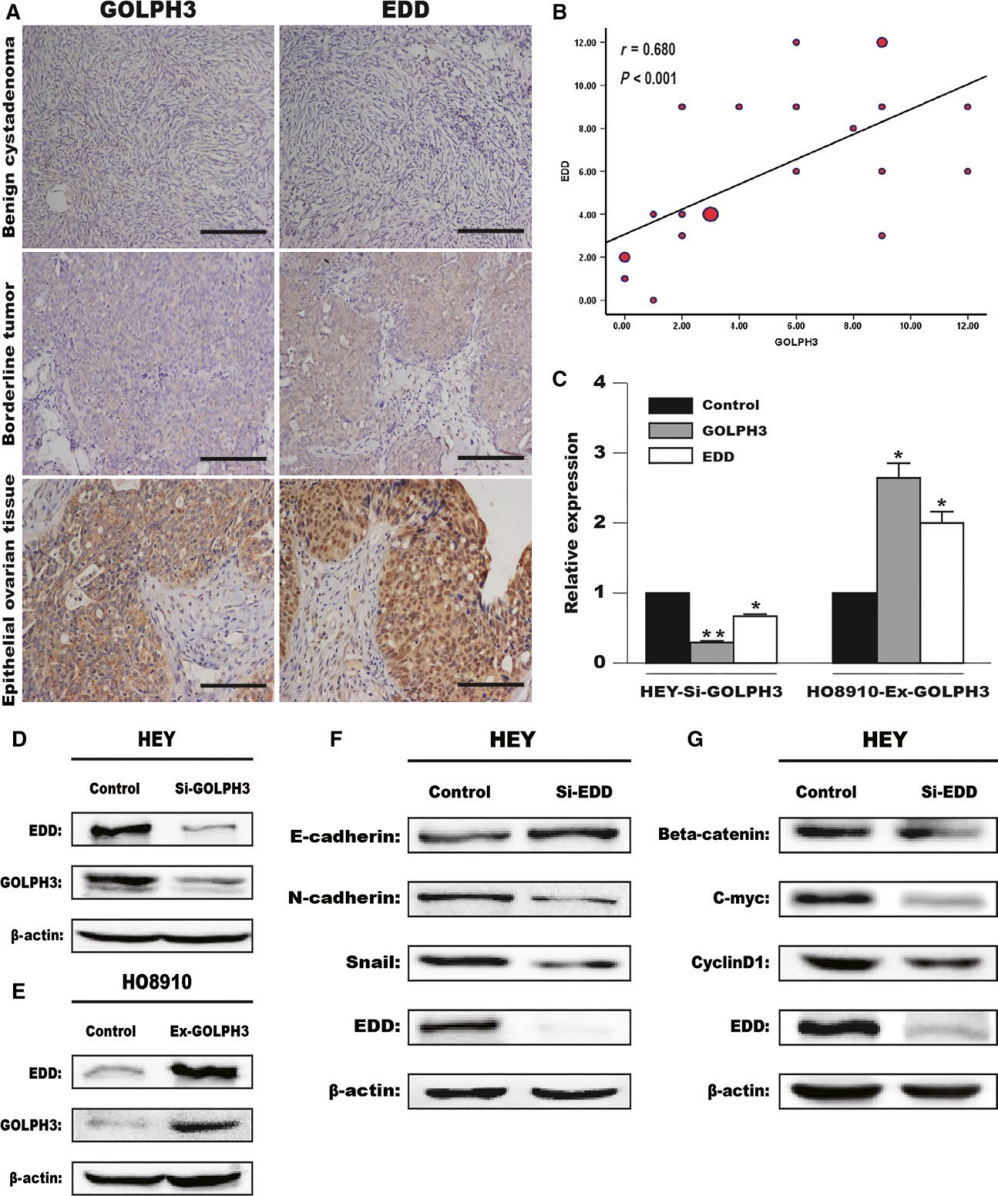
GOLPH3 enhanced EDD expression during Wnt signaling‐induced EMT in EOC cells. (A) The expression of GOLPH3 and EDD was measured using immunohistochemistry in the same section of different tissues. Expression of the nuclear protein EDD was consistent with GOLPH3 expression in benign cystadenoma, borderline tumors and epithelial ovarian tissues. Scale bar: 200 *μ*m. Original magnification 200×. (B) Correlation analysis between GOLPH3 and EDD expression in ovarian tissues (*r* = 0.680, *P* < 0.001). The dot size represented the number of ovarian tissue, respectively. The mRNA (C) and protein levels (D and E) of GOLPH3 and EDD in treated cells were evaluated by quantitative real‐time PCR and western blot analyses. (F and G) Expression levels of EMT‐related markers and Wnt/*β*‐catenin target genes in low‐expressing EDD cells were measured using western blot analysis. *β*‐Actin served as a loading control. *P < 0.05, **P < 0.01 compared with control cells.

Furthermore, we were also interested in whether there is another factor affecting the function of *GOLPH3* in EOC. Finally, we found that the expression levels of *EDD* were consistent with GOLPH3 in different ovarian tissues using immunohistochemistry assays (Fig. 5A). We examined the expression level of *EDD* or *GOLPH3* in the same section of 23 different tissues. As shown in Table 4, there were 2 of 6 cases of benign tumors, all 2 borderline tumors, and 13 of 15 cases of epithelial ovarian cancer that showed high expression of *EDD* protein (*P* < 0.05), while 1 of 2 borderline tumors and 11 of 15 cases of epithelial ovarian cancer showed high *GOLPH3* expression (*P* < 0.01). Correlation analysis then revealed that a positive correlation existed between *GOLPH3* and *EDD* expression in ovarian tissues (*r *=* *0.680, *P *<* *0.001; Fig. 5B).

**Table 4:** GOLPH3 or EDD expression in different ovarian tissues

Tissue type	Total	GOLPH3		*P* value	EDD		*P* value
		High	Low		High	Low	
Benign tumor	6	0	6	<0.01	2	4	<0.05
Borderline tumor	2	1	1		2	0	
Epithelial cancer	15	11	4		13	2	

An IHC score of <3 was considered low expression and a score of 4–12 was considered high expression.

To further investigate the relationship between *EDD* and *GOLPH3*, HEY or HO8910 cells were transfected with *GOLPH3* siRNA or plasmid separately. Using RT‐PCR and western blot assays, we found that silencing or overexpressing *GOLPH3* could significantly reduce or enhance, respectively, the expression of *EDD* (Fig. 5C–E). Furthermore, we examined the expression of EMT‐related markers (*N‐cadherin*,* E‐cadherin*, and *Snail*) and Wnt signaling‐related genes (*β‐catenin*,* c‐Myc*, and *cyclin D1*) in low‐expressing *EDD* cells (Fig. 5F and G). We found that silencing *EDD* decreased the expression of *N‐cadherin*,* Snail*,* β‐catenin*,* c‐Myc*, and *cyclin D1*, while increasing the level of *E‐cadherin*. These results indicate that *GOLPH3* might regulate *EDD* in the GOLPH3–Wnt/*β*‐catenin–EMT axis.

## Discussion

Tumor formation is a multiple step process that is related to abnormal activity of specific genes and particular pathways 28. Previous studies have demonstrated that *GOLPH3*, a newly recognized oncogene, is associated with poor prognosis and aggressive behavior in EOC tissues 16, 17. In this study, we focused on the underlying molecular mechanism of *GOLPH3* in EOC. We found that *GOLPH3* was overexpressed in EOC cell lines and tissues. Moreover, *GOLPH3* promoted EMT progression by activating Wnt/*β*‐catenin signaling, which further influenced the metastatic capacity of EOC. Furthermore, our results showed that *EDD*, a DNA damage‐related factor and oncogene, might play an important role in the GOLPH3–Wnt/*β*‐catenin–EMT axis of EOC.

*GOLPH3* was originally identified as a member of the trans‐Golgi matrix, which plays critical roles in maintaining Golgi traffic and structure and regulating the cellular response to DNA damage 5, 25. Scott et al. reported that *GOLPH3*, a target of 5p13 amplification, was a potent proto‐oncogene in human cancers 10. In this study, we found that the expression of *GOLPH3* was significantly increased in EOC tissues, as well as in cell lines. Similar results have been reported in several types of cancers 12, 14, 15, which demonstrates the vital role of the oncogene *GOLPH3* in tumorigenesis and metastasis.

Previous studies have verified that Golgi PI(4)P promotes tumor metastasis capacity and increases the EMT process by regulating its effector *GOLPH3* 29, 30. Mengke et al. reported that *GOLPH3* promoted cell migration by driving Golgi reorientation to the leading edge 31. Recently, some scholars have proposed that high levels of *GOLPH3* enhance the migration and invasion in several cancers 12, 32, 33. Based on these findings, we hypothesized that *GOLPH3* might be associated with migration and invasion capability in EOC cells. Transwell and scratch migration analyses showed that knocking down *GOLPH3* significantly decreased the migration and invasion capacity of EOC cells. Moreover, the opposite results were observed in *GOLPH3*‐overexpressing cells, which further confirmed our presumption that *GOLPH3* promoted the metastatic capability of EOC cells. Cancer cells in the mesenchymal state can acquire the ability to migrate to distant tissues and form metastases 34. Thus, EMT is regarded as a critical process in cancer metastasis 18, 19. However, there have been no reports exploring the association between *GOLPH3* and the EMT process. In this study, we found that *GOLPH3* clearly downregulated the expression of *E‐cadherin* and upregulated the expression of *N‐cadherin*,* β‐catenin*, and *Snail* compared with the control group, which demonstrated that *GOLPH3* was a new inducer of EMT. In addition, an increasing number of studies have reported that DNA damage might induce EMT via the TGF‐*β* pathway 35, 36. Thus, our data also provides new insight into the association between the DNA damage response and metastasis.

As an important regulator of EMT, the Wnt signaling pathway plays a crucial role in EOC 23. Tatyana et al. reported that the retromer subunit Vps35, which interacts with *GOLPH3*, influenced Wnt secretion by recycling its receptor Wntless 10, 37. *β‐catenin* regulates the classic Wnt signaling pathway by escaping degradation and translocating into the nucleus. Moreover, when the Wnt/*β*‐catenin pathway is activated, specific target oncogenes, such as *c‐Myc* and *cyclin‐D1*, are aberrantly activated 24. High expression levels of these genes indicate activation of the Wnt/*β*‐catenin pathway, which promotes the invasion and metastasis ability of tumor cells. Based on our findings that *GOLPH3* elevated the expression of *β‐catenin*, we suggest that *GOLPH3* might promote the EMT process by activating the Wnt/*β*‐catenin signaling pathway. As we expected, the results of this study showed that high *GOLPH3* expression enhanced the expression level of *c‐Myc* and *cyclin‐D1*. To further confirm our view, we treated cell lines with XAV939 (an inhibitor of the pathway) or LiCl (an activator of pathway). Our results demonstrated that XAV939 blocked and LiCl enhanced the EMT process induced by *GOLPH3*, which effectively support our hypothesis. In view of previous studies reporting that *GOLPH3* modulated mTOR signaling 10, 26, our study may suggest new ideas for investigating the multiple functions of *GOLPH3*. However, whether *GOLPH3* can regulate mTOR signaling in EOC or whether there is cross‐talk among *GOLPH3*, mTOR, and the Wnt signaling pathway remains to be investigated in future studies.

Hay‐Koren et al. reported that the human hyperplastic discs gene, *EDD*, upregulated the expression and enhanced the stability of *β‐catenin* in colorectal cancer 38. Meanwhile, similar to *GOLPH3*,* EDD*, a newly discovered mediator in DNA damage signaling, was overexpressed and modulated cisplatin resistance in ovarian cancer 39, 40, 41. Based on these findings, we first investigated the relationship between *EDD* and *GOLPH3*. Immunohistochemistry assays showed that the expression of the nuclear protein *EDD* was consistent with *GOLPH3* expression in different types of ovarian tumors. Similar results were presented in the western blot and RT‐PCR assays. Furthermore, using western blotting, we found that *EDD* might promote the EMT process, as well as activate Wnt/*β*‐catenin signaling, which means that *EDD* might be regulated by *GOLPH3* in a GOLPH3–Wnt/*β*‐catenin–EMT axis. However, Ohshima et al. reported that *APC* was upregulated by *EDD*, which inhibited β*‐catenin*, resulting in suppression of Wnt signaling 42. Thus, further studies are needed to confirm the hypothesis, and the molecular mechanisms among *EDD* and *GOLPH3* need to be further evaluated.

In summary, this study provides evidence for the existence of a GOLPH3–Wnt/*β*‐catenin–EMT axis, which promotes the migration and invasion capability of EOC cells. Furthermore, we find that *EDD* may be a downstream factor of *GOLPH3* in this axis. Given that chemoresistant tumor cells have an EMT phenotype 43, whether *GOLPH3* regulates chemoresistance in EOC needs to be investigated in further studies. Furthermore, fully understanding the multiple functions of *GOLPH3* may provide new guidance for developing targeted therapy to treat EOC.

## Conflict of Interest

The authors have no conflict of interest to declare.
